# Unveiling the Surge: A Comprehensive Analysis of E-Scooter-Related Injuries at an Urban Level 1 Trauma Center in Vilnius, Lithuania (2018-2021)

**DOI:** 10.7759/cureus.54616

**Published:** 2024-02-21

**Authors:** Kristupas A Suslavičius, Simonas Utkus, Valentinas Uvarovas, Tomas Sveikata, Sigitas Ryliškis

**Affiliations:** 1 Faculty of Medicine, Medical Academy, Lithuanian University of Health Sciences, Kaunas, LTU; 2 Clinic of Rheumatology, Orthopaedics Traumatology and Reconstructive Surgery, Faculty of Medicine, Vilnius University, Vilnius, LTU

**Keywords:** traumatic injuries, rider education, traffic safety, alcohol-related injuries, injury prevention, scooter sharing programs, e-scooter regulations, injury severity, e-scooter injuries, electric scooters

## Abstract

Background

The surge in electric scooter (e-scooter) adoption in 2019 fueled by sharing platforms has raised safety concerns, leading to an increased incidence of e-scooter-related injuries. Despite regulatory efforts, there has been a notable rise in accidents, prompting a comprehensive investigation. This study conducted at the Republican Vilnius University Hospital (RVUH), a level 1 trauma center, is one of the first in the Baltic States aiming to analyze the causes, severity, and frequency of e-scooter injuries from 2018 to 2021. This research addresses a critical gap in understanding e-scooter safety in the Baltic States, providing valuable insights for informed policy and preventive measures.

Methodology

This retrospective study analyzed e-scooter-related injuries in Vilnius, Lithuania, from April to September during 2018-2021. Data from the RVUH emergency department were examined. Using keywords such as “scooter” and “electric,” relevant cases were extracted from the RVUH electronic health system. Included were individuals, both riders and pedestrians, with clear e-scooter involvement, excluding duplicates, those under 18, and users of other types of scooters. Extracted medical records provided data on demographics, injury specifics, helmet use, alcohol consumption, and more. Trauma severity was assessed through the New Injury Severity Score (NISS) and Abbreviated Injury Scale (AIS). Statistical analysis utilized GraphPad Prism software and Excel, adhering to ethical guidelines with RVUH Bioethics Committee approval.

Results

Over four years, 1,036 e-scooter-related injuries at RVUH revealed a gender-based shift, with males sustaining more injuries. The introduction of rentals in 2019 triggered a 334% surge in injuries compared to 2018. Despite an annual 208% increase from 2018 to 2021, 2021 saw a 710.93% rise. Trauma severity remained consistent, with AIS scores 1 and 2 being prevalent. Non-helmet wearers constituted 97.97%, and soft tissue damage was predominant. Ownership patterns shifted toward rentals (81.15%), reflecting the popularity of sharing platforms. Alcohol influence showed no significant change, but intoxicated patients had a higher surgery rate during four years. Mechanism analysis highlighted tripping as the primary cause. Injury characteristics revealed fractures in 34.56% of cases, primarily affecting upper limbs (53.35%). Soft tissue trauma was prominent (65.44%), with lower limbs being significantly impacted.

Conclusions

The surge in e-scooter injuries demands urgent preventive action. While most injuries are mild, a significant proportion is moderate to severe, even fatal. Inadequate education, lax enforcement, and uneven infrastructure contribute to the risk. Urgent measures, including road maintenance, speed reduction, and mandatory helmet use, are crucial. Clarity in government directives for designated e-scooter areas is vital. Further research is needed to understand the broader impact of informed policymaking and safer urban mobility. Expanding research to other Lithuanian regions would enhance the current study.

## Introduction

Electric scooters (e-scooters) have gained popularity as a prevalent mode of transportation in numerous cities worldwide, providing a convenient and environmentally friendly substitute for automobiles and public transit. According to Statista, the user base in Europe has witnessed a more than 24-fold increase over six years, surging from 2018 to over 41 million users by 2023 [1]. This substantial growth is attributed primarily to the introduction of vehicle-sharing platforms, including the launch of an e-scooter-sharing program in major Lithuanian cities in February 2019. E-scooter-sharing platforms enable users to rent e-scooters on a short-term basis [2]. Typically, these services are accessed through a smartphone app, allowing users to locate and unlock available scooters in their vicinity. Upon completing their journey, users can park the scooter at a designated location and conclude the rental through the app [3]. Fueled by heightened demand, such platforms have gained popularity in recent years by providing a convenient and eco-friendly alternative to traditional types of transportation. Consequently, this mode of transport has become widely accessible as an affordable and expedient means of travel [4].

With the introduction of a new mode of transportation, the establishment of corresponding regulations becomes imperative. This was enacted in April 2019 when the Lithuanian Police Department sanctioned compulsory guidelines for e-scooters. Key provisions to the Road Traffic Act include unaccompanied operation permitted from the age of 14, mandatory helmet usage for individuals under 18, and a recommendation for those over 18. Additionally, a maximum speed limit of 25 km/hour is stipulated, and e-scooters are permitted on both roads and pavements. In prioritizing safety, e-scooter development companies have constrained the maximum speed to 25 km/hour, and rental companies have implemented reminders for riders to wear helmets. Furthermore, in areas of heightened risk within the city center, the speed limit has been reduced to 15 km/hour. The blood alcohol limit is 0.4‰ [5].

Despite the implementation of new regulations governing e-scooters, a discernible surge in their popularity has been evident not only on a global scale but also within Lithuania, notably linked to the prevalence of e-scooter-sharing platforms [6-8]. Compounding this, the proliferation of thousands of e-scooters sharing roadways and pedestrian pathways with traditional vehicles, cyclists, and pedestrians has contributed to a notable rise in the incidence of accidents, necessitating emergency department (ED) visits.

This investigation marks the first of its kind in Lithuania and is among the pioneering efforts in the Baltic States. The research was conducted at the Republican Vilnius University Hospital (RVUH), the largest healthcare facility in the Baltic States. The study aims to conduct a detailed analysis of specific causes leading to e-scooter-related injuries, quantify injury severity through standardized measures such as the New Injury Severity Score (NISS) and the Abbreviated Injury Scale (AIS), most affected anatomical localization, demographic characterization, and assessing the frequency and trends of e-scooter-related injuries at the RVUH from 2018 to 2021. This involves examining changes in injury patterns before (2018) and after (2019) the introduction of e-scooter-sharing platforms in Vilnius. The study also aims to compare each year individually and intends to contribute to the global dialogue on e-scooter safety, facilitating a comprehensive comparison with findings from other urban centers.

## Materials and methods

Study design

We conducted a retrospective study to assess e-scooter-related injuries in Vilnius, the capital of Lithuania, spanning from April 1 to September 30 for the years 2018 through 2021. The analysis included all individuals admitted to the adult ED of the RVUH. As a designated level 1 trauma center, RVUH provides comprehensive round-the-clock care for patients with traumatic injuries and serves as the primary facility for high-level trauma care in southern Lithuania.

Data collection

**Figure 1:**
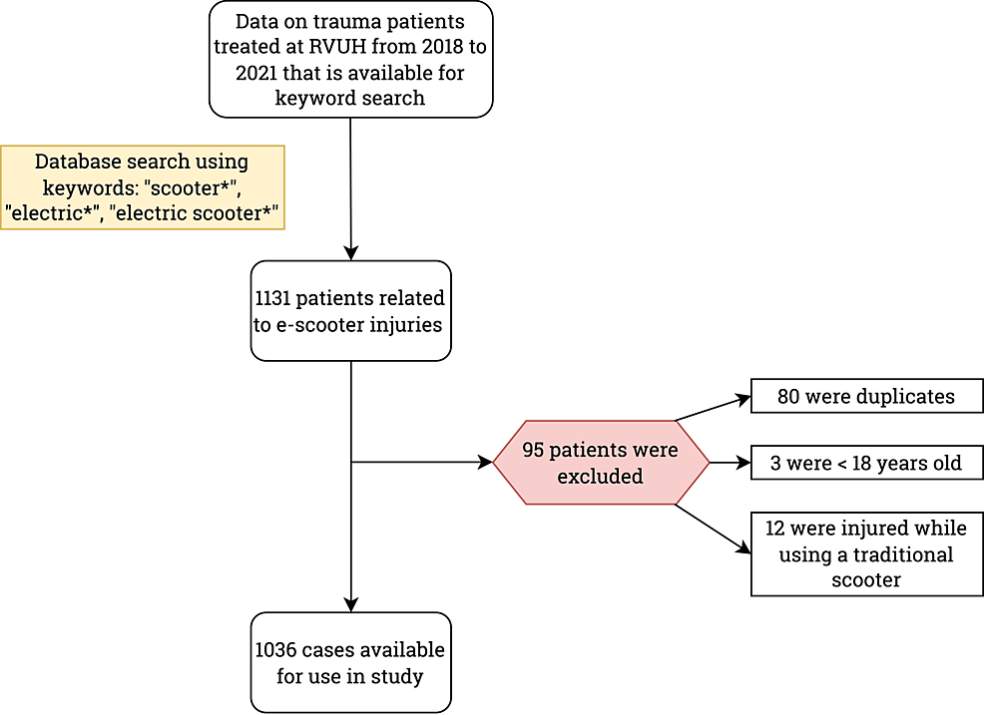
Flowchart of included patients.

An initial data search was conducted to identify patients with injuries related to e-scooters. The information technology (IT) department managing the RVUH electronic health (eHealth) system filtered out ED patients using keywords such as “scooter,” “electric,” and “electric scooter” and their inflected forms. Subsequently, the medical records of selected patients were extracted and depersonalized by the RVUH IT department, ensuring inclusion only of cases where the involvement of e-scooters was unequivocal. Patients presenting to the ED with e-scooter-related injuries, either as riders or pedestrians, were included, while duplicates, individuals younger than 18 years, and those driving mopeds, Segways, or traditional scooters were excluded (Figure 1). Data extracted from medical records included information on the date, location, severity, injury outcomes, age, gender, helmet use, alcohol consumption, speed, e-scooter ownership, and operations performed due to the injury. Trauma severity was assessed by RVUH traumatologists using the NISS and AIS [9,10]. According to AIS, minor injuries encompassed superficial wounds, muscle or ligament sprains, mild contusions, dental injuries, and mild concussions without loss of consciousness or equivalent. Moderate injuries included closed fractures of distal extremities, major ligament tears (e.g., knee cruciate ligament tear), and their equivalents. Serious injuries were classified as open fractures, fractures of the proximal femur, comminuted facial fractures, basilar skull fractures, and intracranial hemorrhages with a coma duration of less than six hours. The NISS was calculated by assessing the AIS score for six distinct body regions (head or neck, face, chest, abdominal or pelvic contents, extremities or pelvic girdle, and external areas) and summing the squared values of the top three scores among these regions. In addition, injuries were categorized as bone fractures or soft tissue damage. Injury locations were specified as head, face, neck, upper abdomen, lower abdomen, shoulder, upper limb (including upper and lower arm, elbow, wrist, hand, and fingers), and lower limb (including upper and lower leg, knees, ankle, foot, and toes). The mechanism of injury was classified as rider and non-rider injuries. Rider injuries included falling (e.g., tripping over uneven roads, slippery surfaces, or two persons falling on the same scooter) and collisions (e.g., with an object, moving car, or another scooter or bike), as well as an e-scooter malfunction. Non-rider injuries were categorized as being hit by a scooter and tripping over a scooter on the road.

Data analysis

Statistical analysis was conducted utilizing GraphPad Prism software version 9.5.1 (San Diego, CA, United States) and Excel version 16.82 (Microsoft Corporation, Redmond, WA, United States). Data normality was assessed through the Shapiro-Wilk and Kolmogorov-Smirnov tests. Descriptive statistics were presented as mean (±) and median values (minimum-maximum) for continuous variables, and frequency with percentages for categorical variables. Continuous variables were subjected to analysis of variance, Kruskal-Wallis, Mann-Whitney U tests, and post hoc analyses, including Dunnett’s and Dunn’s tests. Categorical variables were assessed using the Pearson chi-square test or Fisher’s exact test. All statistical analyses employed two-tailed tests, and the level of statistical significance was set at 0.05. The study’s findings were visually presented through tables and figures.

Ethics approval

The research obtained approval from the Bioethics Committee at the RVIH on August 9, 2022, under approval number 2R-5.4.-4098. The study was grounded in an anonymous registry, consequently exempting the requirement for informed consent. The research strictly adhered to relevant guidelines and regulations in alignment with the principles outlined in the Declaration of Helsinki. The data were stored on a secure computer in the Orthopedics and Traumatology Center at RVUH Hospital.

## Results

We identified 1,036 e-scooter-related injuries treated at RVUH from 2018 to 2021 during the season from April to September. There were 417 (40.3%) females and 619 (59.7%) males. Statistically significant differences were observed in the distribution of injuries between males and females in 2019, 2020, and 2021, with more males sustaining injuries than females (p = 0.002, p < 0.001, p < 0.001, respectively). However, in 2018, there was no significant difference in injury distribution between males and females (p > 0.05). Across the four years, there was no statistically significant difference observed among the age groups, and the predominant age range for the majority of patients was between 18 and 30 years. The median age for all years combined was 31 (18-87) years. When comparing the sexes individually across all years, the median age for men was 31 (18-87) years, and for women, it was 30 (18-74) years, with no statistical difference identified (p = 0.448) (Table 1).

**Table 1:** Demographic characteristics of e-scooter drivers.

Year	2018 (n = 64)	2019 (n = 214)	2020 (n = 303)	2021 (n = 455)	Total (n = 1,036)	
Variable	n (%)	n (%)	n (%)	n (%)	n (%)	P-value
Age, median (range)	30.5 9 (19-67)	31 (18-87)	30 (18-80)	30 (18-70)	31 (18-87)	
Age groups						
18-30	29 (45.31)	88 (42.31)	131 (44.71)	208 (47.49)	456	0.711
30-39	18 (28.13)	73 (35.10)	96 (32.76)	127 (29)	314	0.374
40-49	9 (14.06)	23 (11.06)	36 (12.29)	61 (13.92)	129	0.755
50-59	5 (7.81)	16 (7.69)	15 (5.12)	28 (6.39)	64	0.636
>60	3 (4.69)	8 (3.85)	15 (5.12)	14 (3.2)	40	0.604
Gender						
Male	34 (52.13)	123 (57.48)	176 (58.09)	286 (62.86)	619	0.284
Female	30 (46.87)	91 (42.52)	127 (41.91)	169 (37.14)	417	

**Figure 2:**
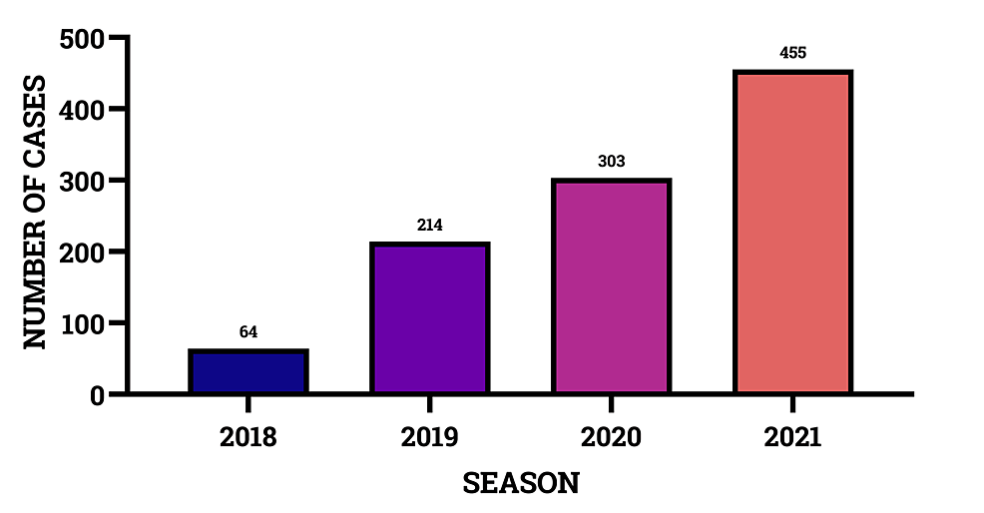
Electric scooter-related trauma occurrence from 2018 to 2021.

In 2019, with the introduction of e-scooter rentals, there was a 334% increase in injured riders compared to 2018. From 2018 to 2021, there was an annual 208% increase. In 2021, there was a 710.93% increase in injured e-scooter drivers compared to 2018 (Figure 2).

When comparing trauma severity across four years, there was no statistically significant difference, with minor severity (AIS 1-8) being the most common across all years. Additionally, the AIS score distribution indicated that AIS scores 1 and 2 were consistently the most prevalent in all four years. However, a statistically significant difference was observed in AIS score 2 (moderate) between years (p = 0.011). In 2018, there were significantly more injured patients with AIS score 2 (34.38%) than in 2020 (18.15%) and 2021 (20%) (p = 0.1737, p = 0.0091, respectively), with no significant difference observed in 2019 (p = 0.1737) (Table 2). The two reported deaths occurred within the 50-59-year age interval and were associated with alcohol use and fatal/critical AIS levels. Both deaths were linked to computed tomography (CT)-verified severe brain damage and were not associated with helmet use.

**Table 2:** Trauma severity. NISS: New Injury Severity Score; AIS: Abbreviated Injury Score

Year	2018 (n = 64)	2019 (n = 214)	2020 (n = 303)	2021 (n = 455)	Total (n = 1,036)	
Variable	n (%)	n (%)	n (%)	n (%)	n (%)	P-value
NISS						
Minor (1-8)	57 (89.06)	190 (88.79)	267 (88.12)	383 (84.2)	897 (86.58)	0.2491
Moderate (9-14)	7 (10.94)	19(8.88)	32 (10.56)	67 (14.81)	125 (12.07)	0.1215
High (≥15)	0 (0)	5 (2.34)	4 (1.32)	5 (0.99)	14 (1.35)	0.4481
AIS						
1 (minor)	35 (54.69)	135 (63.08)	212 (69.97)	292 (64.18)	674 (65.06)	0.0799
2 (moderate)	22 (34.38)	55 (25.70)	55 (18.15)	91 (20)	223 (21.53)	0.011
3 (serious)	7 (10.94)	20 (9.35)	34 (11.22)	69 (15.16)	130 (12.55)	0.1401
4 (severe)	0 (0)	4 (1.87)	1 (0.33)	2 (0.44)	7 (0.68)	0.1168
5 (critical)	0 (0)	0 (0)	0 (0)	1 (0.22)	1 (0.10)	0.7343
6 (fatal)	0 (0)	0 (0)	1 (0.33)	0 (0.00)	1 (0.10)	0.4896

When comparing outcomes, the most common injury type was soft tissue damage in all four years (62.61% in total) with no statistically significant difference when compared to bone fractures. When examined yearly, the statistical analysis revealed a significant prevalence of non-helmet wearers compared to helmet wearers in each year (p < 0.001), resulting in a total of 97.97% (n = 1,015) non-helmet wearers. When comparing the injury severity assessed by AIS between individuals wearing helmets (n = 21) and those not wearing helmets (n = 1,015), no statistically significant differences were observed in AIS scores 1, 2, 3, 4, 5, and 6 (p = 0.1227, p = 0.523, p = 0.5018, p > 0.9999, p > 0.9999, p > 0.9999, respectively). The most prevalent severity level was AIS score 1, with a frequency of 80.95% (n = 17) among helmet wearers and 64.73% (n = 657) among non-helmet wearers. Among the individuals who wore a helmet, none exhibited subarachnoid hemorrhages or epidural hematomas, while one case was diagnosed with a concussion. The operation rate due to e-scooter injuries was 12.26% (n = 127) of all patients, and there was no statistically significant difference between the years. When comparing those who required surgery, each year, a higher number of operations for e-scooter injuries were performed on men (63.77%) than on women, with no statistically significant difference between the years (p = 0.96) (Table 3).

**Table 3:** Accident characteristics. n/a: not assessed

Year	2018 (n = 64)	2019 (n = 214)	2020 (n = 303)	2021 (n = 455)	Total (n = 1,036)	
Variable	n (%)	n (%)	n (%)	n (%)	n (%)	P-value
Outcomes						
Bone fracture	29 (45.31)	72 (33.64)	93 (30.69)	169 (37.39)	358 (34.55)	0.080
Soft tissue damage	35 (54.69)	142 (66.36)	210 (69.31)	283 (62.61)	678 (65.44)	
Helmet use						
Yes	1 (1.56)	4 (1.87)	7 (2.31)	9 (1.98)	21 (2.03)	0.974
No	63 (98.44)	210 (98.13)	296 (97.69)	446 (98.02)	1,015 (97.97)	
Surgery due to injury	8 (12.5)	23 (10.75)	34 (11.22)	62 (13.63)	127 (12.26)	0.670
Surgery due to injury between genders						
Women	3 (37.5)	9 (39.13)	13 (38.24)	21 (33.87)	46 (36.22)	0.960
Men	5 (62.5)	14 (60.87)	21 (61.76)	41 (66.13)	81 (63.77)	
Ownership of e-scooter						
Rental	0 (0)	157 (84.86)	221 (85.99)	367 (89.08)	745 (81.15)	<0.0001
Own	64 (100)	28 (15.14)	36 (14.01)	45 (10.92)	173 (18.85)	
Alcohol intoxication						
Positive	3 (4.69)	13 (6.07)	19 (6.27)	37 (8.13)	72 (6.95)	0.581
Negative	6 (9.38)	23 (10.75)	29 (9.57)	45 (9.89)	103 (9.94)	0.973
Unknown	55 (85.94)	178 (83.18)	255 (84.16)	373 (81.98)	861 (83.11)	0.797
Speed at time of injury, median (range)	n/a	n/a	n/a	15 (8-30)	n/a	n/a

When examining e-scooter ownership, a statistically significant difference was observed between years (χ² = 298.1, p < 0.0001). In 2018, all patients were using their e-scooters, as the sharing function had not yet been introduced. Comparing 2019 to 2021, the majority of injured patients significantly were utilizing rented rather than own e-scooters (p < 0.001). Overall, 81.15% (n = 745) of all patients were using rentable e-scooters (Table 3).

When comparing patients who were intoxicated with alcohol, the majority of data was unknown (83.11% in total). Subsequent results were based only on known information. There was no statistically significant difference between intoxicated and sober drivers over the four years (χ² (3, N = 175) = 1.187, p = 0.756); the majority of patients (58.86%) were sober. Significantly more intoxicated patients (72.58%) required surgery compared to sober patients (37.86%) (χ² = 18.66, p < 0.0001). In the years 2019-2021, among those who were under the influence of alcohol (n = 72 in total), 92.75% (n = 64) utilized a rented e-scooter at the time of the injury, with no significant difference between the years (p = 0.454) (Table 3).

The median speed at the time of injury was only known in 2021, and it was determined to be 15 km/hour with a range of 8 km/hour to 30 km/hour (Table 3).

Among those who suffered injuries, 92.28% (n = 956) were e-scooter drivers, while 7.72% (n = 80) were pedestrians, with no statistically significant difference observed between the years. Furthermore, there was no statistically significant difference in the mechanisms of injury over the four-year period. Tripping over uneven road surfaces emerged as the most frequent cause of injury in all of the cases across all years: 2018 (57.81%), 2019 (47.2%), 2020 (53.63%), and 2021 (51.25%). The second most common cause throughout all four years was a collision with an object: 2018 (21.88%), 2019 (21.96%), 2020 (18.24%), and 2021 (19.98%). When comparing only non-riders, the most common cause of the injury was being hit by a scooter (86.25%) (Table 4).

**Table 4:** Mechanism of the injury.

Year	2018 (n = 64)	2019 (n = 214)	2020 (n = 303)	2021 (n = 455)	Total (n = 1,036)	
Variable	n (%)	n (%)	n (%)	n (%)	n (%)	P-value
Rider injured	62 (96.88)	200 (93.46)	280 (92.41)	414 (90.99)	956 (92.28)	0.336
Rider fell	46 (71.88)	138 (64.49)	198 (65.35)	307 (67.47)	689 (66.51)	0.661
Tripped over uneven road surface	37 (57.81)	101 (47.20)	149 (49.17)	244 (53.63)	531 (51.25)	0.255
Slippery road surface	6 (9.38)	21 (9.81)	27 (8.91)	38 (8.35)	92 (8.88)	0.939
Two persons fell on the same scooter	0 (0.00)	4 (1.87)	8 (2.64)	10 (2.20)	22 (2.12)	0.603
Failure to control	3 (4.69)	12 (5.61)	14 (4.62)	15 (3.30)	44 (4.25)	0.548
Collision	15 (23.44)	54 (25.23)	76 (25.08)	100 (21.98)	245 (23.65)	0.718
Collision with an object	14 (21.88)	47 (21.96)	63 (20.79)	83 (18.24)	207 (19.98)	0.647
Collision with a moving car	1 (1.56)	4 (1.87)	7 (2.31)	9 (1.98)	21 (2.03)	0.974
Collision of a scooter into a scooter or bike	0 (0.00)	3 (1.40)	6 (1.98)	8 (1.76)	17 (1.64)	0.706
E-scooter malfunction	1 (1.56)	8 (3.74)	6 (1.98)	7 (1.54)	22 (2.12)	0.313
Non-rider injured	2 (3.13)	14 (6.54)	23 (7.59)	41 (9.01)	80 (7.72)	0.336
Hit by a scooter	2 (3.13)	12 (5.61)	19 (6.27)	36 (7.91)	69 (6.66)	0.409
Tripped over scooter on the road	0 (0.00)	2 (0.93)	4 (1.32)	5 (1.10)	11 (1.06)	0.821

In total, 59.65% (n = 618) of all patients sustained multiple injuries, including fractures and soft tissue damage. The most frequently injured region was the upper limb in 2018 (37.76%), 2019 (39.7%), and 2021 (34.1%), whereas, in 2020, the lower limb was the most affected (34.3%). Analyzing the most injured specific anatomical localization, the head accounted for 13.27% in 2018, the hand and fingers for 15.5% in 2019, and the lower arm for 11% in 2020 and 11.7% in 2021. Statistically significant differences between years were observed in the upper arm (χ² = 13.84, p = 0.0031), elbow (χ² = 10.37, p = 0.0157), wrist (χ² = 10, p = 0.0185), lower limb (χ² = 14.49, p = 0.0023), upper leg (χ² = 19.08, p = 0.0003), and knee (χ² = 7.979, p = 0.0464). In 2021, upper arm injuries were significantly higher (4.81%) compared to 2019 (0.88%) (p = 0.0012) and 2020 (2.37%) (p = 0.028), with no significant difference observed in comparison to 2018 (p = 0.2987). Notably, there were fewer elbow injuries in 2021 (5.23%) than in 2018 (11.22%) (p = 0.0189) and 2019 (7.89%) (p = 0.0057), with no significant difference observed in 2020 (p = 0.0611). Regarding wrist injuries, a statistically significant increase was observed in 2021 (5.23%) compared to 2019 (1.75%) (p = 0.0077), while no significant difference was found between 2020 (2.96%) and 2018 (2.04%). Analysis of lower limb injuries revealed a statistically significant increase in 2020 (34.32%) compared to 2019 (23.1%) (p = 0.0005) and 2021 (27.44%) (p = 0.0101), with no significant difference observed in comparison to 2018 (24.49%). In the upper leg, there were significantly more fractures in 2021 (4.53%) compared to 2018 (0%) (p = 0.0247), 2019 (0.88%) (p = 0.0014), and 2020 (1.58%) (p = 0.005). Additionally, statistically more knee injuries occurred in 2020 (9.47%) compared to 2021 (5.37%) (p = 0.0061), with no significant difference observed in comparison to 2018 (6.12%) and 2019 (6.43%) (Table 5).

**Table 5:** Injury characteristics based on anatomical localization.

Year	2018 (n = 64)	2019 (n = 214)	2020 (n = 303)	2021 (n = 455)	Total (n = 1,036)	
Variable	n (%)	n (%)	n (%)	n (%)	n (%)	P-value
Head	13 (13.27)	34 (9.94)	43 (8.48)	56 (7.92)	146 (8.83)	0.2929
Face	8 (8.16)	34 (9.94)	40 (7.89)	72 (10.1)	154 (9.31)	0.5313
Neck	0 (0.00)	6 (1.75)	2 (0.39)	6 (0.85)	14 (0.85)	0.1429
Upper abdomen	3 (3.06)	13 (3.80)	23 (4.54)	42 (5.94)	81 (4.90)	0.3359
Lower abdomen	2 (2.04)	3 (0.88)	15 (2.96)	13 (1.84)	33 (2.00)	0.1966
Shoulder	11 (11.22)	37 (10.8)	37 (7.30)	58 (8.20)	143 (8.65)	0.2396
Upper limb	37 (37.76)	136 (39.7)	173 (34.1)	266 (37.6)	612 (37.00)	0.3816
Upper arm	2 (2.04)	3 (0.88)	12 (2.37)	34 (4.81)	51 (3.08)	0.0031
Elbow	11 (11.22)	34 (9.94)	40 (7.89)	37 (5.23)	122 (7.38)	0.0157
Lower arm	11 (11.22)	40 (11.7)	56 (11.0)	83 (11.7)	190 (11.49)	0.9833
Wrist	2 (2.04)	6 (1.75)	15 (2.96)	37 (5.23)	60 (3.63)	0.0185
Hand and fingers	11 (11.22)	53 (15.5)	50 (9.86)	75 (10.6)	189 (11.43)	0.063
Lower limb	24 (24.49)	79 (23.1)	174 (34.3)	194 (27.4)	471 (28.48)	0.0023
Upper leg	0 (0.00)	3 (0.88)	8 (1.58)	32 (4.53)	43 (2.60)	0.0003
Knees	6 (6.12)	22 (6.43)	48 (9.47)	38 (5.37)	114 (6.89)	0.0464
Lower leg	2 (2.04)	19 (5.56)	43 (8.48)	42 (5.94)	106 (6.41)	0.0596
Ankle	11 (11.22)	19 (5.56)	40 (7.89)	48 (6.79)	118 (7.13)	0.2285
Foot and toes	5 (5.10)	16 (4.68)	35 (6.90)	34 (4.81)	90 (5.44)	0.3818

**Figure 3:**
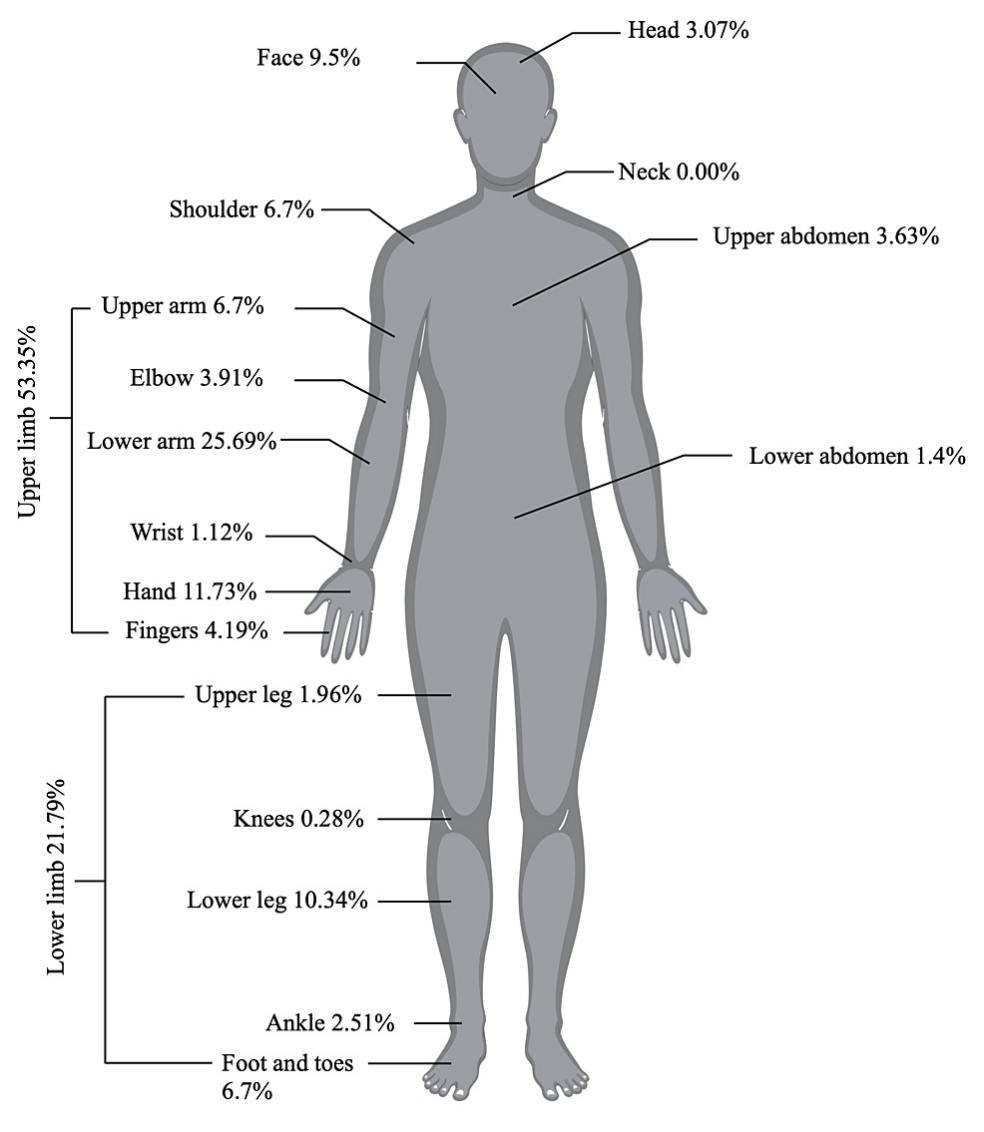
Fracture location of all patients.

Out of the total 1,036 reported cases, 34,56% (n = 358) resulted in fractures. More than half of these fractures affected the upper limb (53.35%), comprising mostly ulnar and radial fractures (25.7%), followed by fractures of the hand bones (11.73%). Approximately one-fifth of the fractures were of the lower limb (21.79%), specifically affecting the tibia and fibula (10.34%). Facial fractures were also relatively common, accounting for 9.5% (Figure 3). Two people got injured due to an e-scooter malfunction-caused crash (one brake failure, and one steering wheel handlebar failure). Patients who experienced the malfunctions only suffered lower limb soft tissue damage (with both AIS and NISS equal to 1).

**Figure 4:**
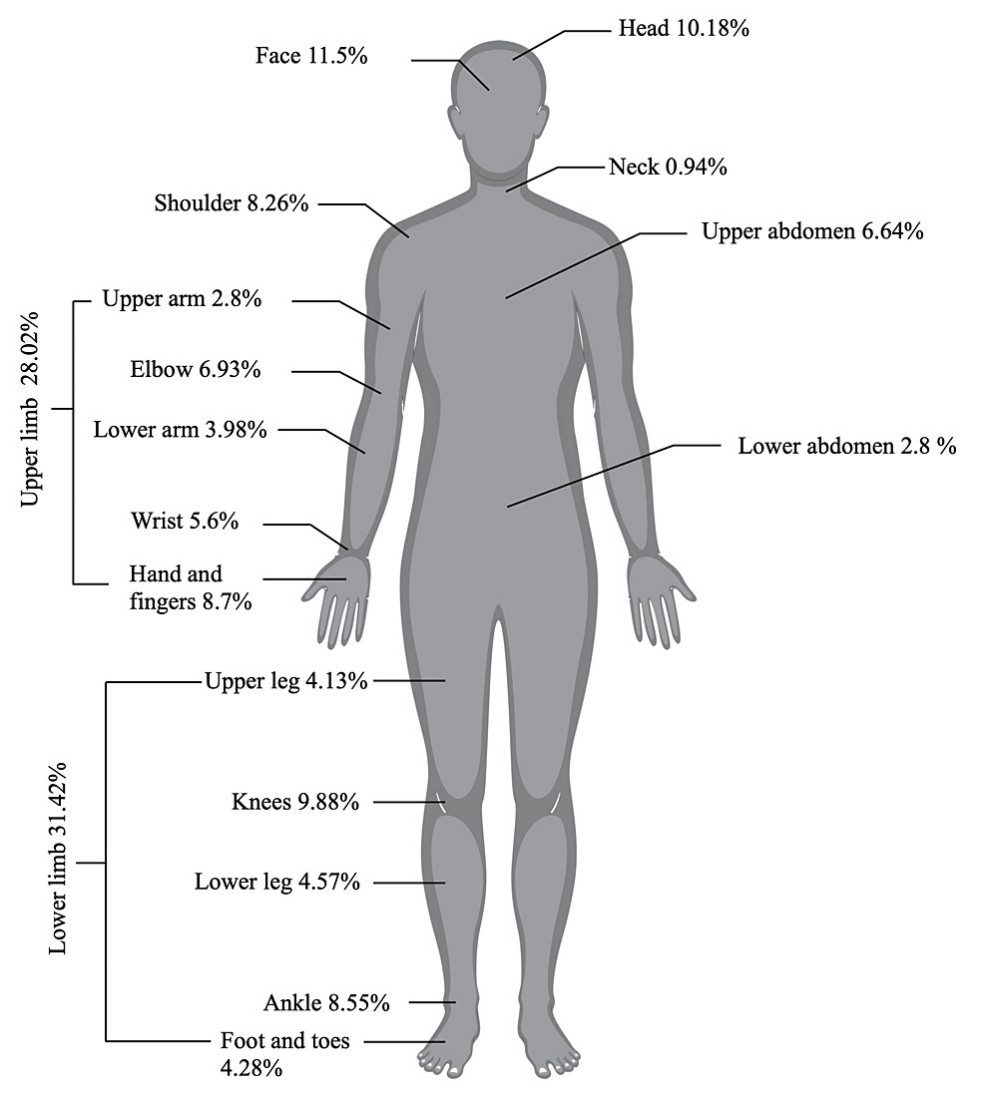
Soft tissue trauma location of all patients.

Of the total 1,036, 65.44% (n = 678) patients experienced only soft tissue trauma, such as lacerations, abrasions, and contusions. Among these, the lower limbs exhibited the most pronounced impact, affecting 31.42%, with the knee (9.88%) and ankle (8.55%) being the most affected sites. Slightly over a quarter of patients experienced upper limb injuries (n = 190; 28.02%), with the hand and fingers (8.7%) and elbow (6.93%) being the predominant areas of concern. Notably, the face (11.5%) and head (10.18%) were frequently affected in the patient population (Figure 4).

## Discussion

In the wake of the worldwide deployment of e-scooter-sharing services, researchers globally have disseminated disconcerting findings about injuries associated with e-scooters [7,11-13]. Consequently, predicated on anecdotal insights, antecedent to the commencement of our study, a prevailing conjecture emerged that the incidence of such injuries in Lithuania would likely parallel or potentially surpass those reported elsewhere. This speculation prompted the imperative need for a dedicated study to elucidate these conjectures, allowing for nuanced comparisons between the Lithuanian dataset and analogous data sourced from diverse international locales. Anticipating a dearth of comprehensive investigations into this domain within Lithuania, our study embarked on uncharted territory. A meticulous exploration of existing literature fortifies our belief that this research endeavors to be the inaugural inquiry of its nature within the Lithuanian landscape, marking a pioneering initiative among the Baltic States. The overarching goal is to contribute significantly to the collective understanding of the implications of e-scooter usage, providing a foundation for informed policymaking and public safety measures in the region.

Unsurprisingly, our investigation, alongside pertinent antecedent studies, substantiates a discernible surge in the incidence of patients seeking emergency care following the implementation of e-scooter-sharing services, manifesting not only across Europe but globally. Notably, in Lithuania, after the initiation of the aforementioned e-scooter rental program, the prevalence of e-scooter-related injuries exhibited a substantial annual escalation averaging 208%. Despite the introduction of rental e-scooters and the elevated incidence of e-scooter injuries documented in 2019, the subsequent reduction in speed to 15 km/hour on certain Vilnius streets from the beginning of 2020 has not resulted in a decline in the escalating number of injuries. The most notable surge occurred between 2018 (pre-dating the rental program) and 2021, reaching an unprecedented pinnacle of 710.93%. Analogous trends were discerned in the United States, where the annual count of e-scooter-related injuries skyrocketed by 222% between 2014, a period devoid of scooter ride-share companies, and 2018, amassing 14,651 incidents. A congruent escalation was observed in Tel Aviv, Israel, reflecting a sixfold increase [6,7,14]. Vienna, Austria, experienced an even more pronounced surge, registering an 892% elevation in e-scooter-associated injuries [15]. New Zealand, too, faced a surge in injury rates, with researchers noting an escalation from two injuries per week before the introduction of e-scooter-sharing services to an average of 35 injuries per week post-implementation between September 2018 and April 2019 [16]. The burgeoning popularity of e-scooter-sharing platforms, while transformative in urban mobility, has exacted a toll - an alarming upswing in associated injuries. Our empirical findings corroborate the observations made by numerous studies, underscoring a consistent and escalating pattern in injury rates concomitant with the proliferation of electric scooter-sharing platforms. This confluence of evidence underscores the imperative for a comprehensive understanding of the multifaceted safety challenges posed by these platforms, necessitating concerted efforts in injury prevention and public safety measures.

Within the scope of our investigation, a noteworthy observation pertains to the prevalence of fractures among e-scooter-related injuries, with 34.56% of cases exhibiting this outcome. Strikingly, this finding closely parallels the results of a retrospective cohort study conducted in Helsinki, where the incidence of fractures was reported at 34% [17]. This convergence underscores a remarkable consistency in the fracture rates across different geographic contexts, thereby substantiating the global pattern of injury occurrences associated with e-scooter usage. Further delving into the realm of treatment modalities, our study revealed that operative interventions were necessitated in 12.26% of cases. This proportion aligns closely with the operative treatment rate of 12% documented in the aforementioned Helsinki study, highlighting a comparable demand for surgical interventions following e-scooter-related injuries in disparate locations [17]. To contextualize our findings within the broader landscape of existing literature, we turn to systemic reviews encompassing a collective analysis of 34 studies. This comprehensive examination elucidated a fracture incidence of 39.2%, with a notable subset of cases, comprising 17.2%, mandating surgical interventions. A scoping review, aggregating insights from 28 studies and scrutinizing injury types, unveiled that almost one-third of patients, precisely 30.7% (range: 11.5%-70.5%), sustained fractures [18,19]. These synthesized outcomes accentuate the pervasive nature of fractures in the spectrum of e-scooter-related injuries, portraying a consistent narrative across diverse studies and methodologies. In alignment with antecedent research, our study affirms the upper extremity as the predominant anatomical site of injury. This recurrent pattern is echoed in the existing literature, with some investigations positing the head as the body part most frequently impacted during falls. The congruence in these findings underscores the anatomical vulnerability associated with e-scooter accidents, emphasizing the need for targeted preventive measures focused on these specific regions.

The recent surge in e-scooter utilization has prompted heightened apprehensions concerning safety, extending beyond the well-being of riders to encompass pedestrians. Pedestrians affected by e-scooter incidents encounter comparable potential financial ramifications stemming from hospitalization costs, medical interventions, work absenteeism, and rehabilitative therapies, akin to the burdens faced by riders. Despite these concerns, the existing body of literature inadequately addresses the impact of e-scooters on pedestrians. Empirical evidence from prior studies indicates that individuals particularly susceptible to injuries arising from pedestrian transportation include those with visual and/or auditory impairments, young children, the elderly, and individuals distracted by mobile devices [20]. Noteworthy is the consensus among riders themselves regarding the heightened risk of collisions, with half of e-scooter riders reporting near misses, over 50% of which involve interactions with other road users [21]. Literature indicates that non-riders most frequently sustain injuries either by being struck by an e-scooter (59.1%) or by tripping over a parked e-scooter (29.5%). When comparing various injury types, e-scooter-related trauma sustained by non-riders comprises approximately 2% of the total [17,19]. Improperly parked shared e-scooters obstruct sidewalks and pose hazards to pedestrians, potentially causing stumbling incidents or compelling them to use roads to circumvent blockages, thereby heightening the risk to pedestrians [21]. A preponderance of pedestrians (86%) reportedly experience relatively minor injuries that do not necessitate hospital admission [19]. In consonance with our study outcomes, our investigation identified 11 pedestrians (13.75% of all pedestrian trauma) who incurred injuries while tripping over e-scooters, while 69 pedestrians (86.25%) were struck by e-scooters. Notably, these incidents constituted just under 8% of all cases presenting at the ED, characterized by comparably minor injuries that did not warrant hospitalization.

Numerous factors contribute to the occurrence of patients falling from e-scooters, warranting in-depth analysis. Primarily, the design and structural characteristics of these scooters significantly amplify the susceptibility to accidents and falls. Additionally, user behavior and insufficient training play pivotal roles in contributing to such incidents. Furthermore, external elements, such as unfavorable road conditions and inadequate infrastructure, exacerbate the likelihood of patients encountering accidents while using electric scooters. A comprehensive understanding of these factors is imperative for delineating measures aimed at mitigating the risk of falls and enhancing the safety of patients utilizing these devices. Systemic reviews indicate that 86.8%-92.8% of riders experience injuries in single road user events, while 7.1%-13.3% sustain injuries in multiple road user events. Single-user incidents encompass falls, collisions with objects, excessive speed, and adverse road conditions, with falls representing the most prevalent category, ranging from 74.4% to 94.6% [19]. Aligning with these patterns, our study revealed that 66.51% of riders experienced falls, positioning this outcome precisely within the midpoint of the spectrum identified in systemic reviews. This alignment underscores the consistency between our findings and existing literature, reinforcing the predominance of falls as a primary mechanism of injury among e-scooter users.

Upon scrutinizing safety measures among cases documented at RVUH, our findings indicate a concerning trend, with helmet usage observed in fewer than 3% of e-scooter riders. This conspicuous deficiency in helmet use is consistently pervasive across various studies, with the majority reporting rates ranging from 2% to 5% [6,8,19,22,23]. Notably, the reported results exhibit a wide range, spanning from as low as 0% to a substantial 46% [18]. Studies posit that these extremes are largely influenced by the status of helmet laws [16]. The landscape of helmet adoption presents a nuanced scenario in many countries, such as Lithuania, where helmet wear is merely recommended, resulting in a gradual or stagnant growth in the percentage of helmet wearers. Significantly, our study has revealed that predominant injuries occur during the use of e-scooters rented from rental companies, which do not offer any physical protection measures, including helmets. It is imperative to acknowledge that the lack of comprehensive documentation, particularly concerning helmet type (half-face or full-face) and usage in numerous injury cases and related literature, introduces ambiguity, compromising result precision. Aligning with the recommendations of fellow researchers, we advocate for additional documentation regarding safety measures in routine clinical settings. Furthermore, the proposition to implement helmet laws warrants serious consideration. Empirical evidence underscores the substantial impact of helmet use in mitigating craniofacial trauma, as supported by existing studies [24,25]. In our study, individuals who wore helmets exhibited no instances of subarachnoid hemorrhage and epidural hematoma. The two reported fatalities were directly associated with severe head trauma and a lack of helmet use. Acknowledging the retrospective nature of our study, we express regret over the inability to collect comprehensive information regarding the utilization of additional safety gear. This limitation underscores the imperative for future studies to address these gaps and augment our understanding of the broader safety measures adopted by e-scooter riders.

A key factor influencing injuries is the speed at which e-scooters operate, a familiar element for urban dwellers. Understanding the implications of speed and velocity is crucial, as it directly correlates with the magnitude and severity of trauma associated with e-scooter use. While our data collection was limited to 2021, we posit that individuals sustaining serious injuries experience considerably higher speeds during trauma events compared to those not requiring urgent care. From the subjective perspective of patients, the median speed at the time of injury hovered around 15 km/hour, with a range spanning from 8 km/hour to 30 km/hour. These findings parallel those of a discreet speed-tracking device, which reported an average speed of 15.4 km/hour in Trondheim, Norway [26]. The literature suggests a connection between speed and the escalating incidence of injuries. In Helsinki, Finland, researchers observed a substantial reduction in injury rates by lowering daytime speed from 25 to 20 km/hour and limiting nighttime top speed to 15 km/hour [27]. Conversely, some researchers argue that scooter speed may not be directly associated with the risk of hospitalization and biomechanically may be less crucial than factors such as the angle of approach to vertical surfaces, such as curbs or objects [28]. Certain cities, like Singapore, have adopted lower speed limits, restricting e-scooters to 10 km/hour. This not only aligns with safety measures but also promotes a sense of security among pedestrians, as suggested by scientists from Singapore [29]. Initiating in September 2023, Vilnius, Lithuania, implemented analogous measures, capping the maximum speed of e-scooters in the old town at 12 km/hour. These speed restrictions were automatically enforced by all scooter-sharing companies [30]. However, a notable discrepancy exists between rental scooter companies, enforcing speed limits below 20 km/hour, and privately owned electric scooters, which operate without such restrictions and often escape penalties for exceeding speed limits. Consequently, we emphasize the urgent need for extensive investigation into the relationship between e-scooter speed and trauma incidence and severity. Until such research unfolds, emulating speed-limiting strategies from other cities represents a prudent choice, alleviating the strain on local urgent care facilities.

Road conditions play a pivotal role in the safety of all road users, especially those operating e-scooters. The alarming increase in the popularity of e-scooters in recent years has brought attention to the potential dangers associated with inadequate road conditions. It is crucial to acknowledge how these conditions contribute to higher injury rates among e-scooter riders. The findings obtained through real-world riding experiments reveal that e-scooters are significantly affected by various riding conditions. In particular, riding e-scooters involved more intense occurrences of vibrations in comparison to riding bicycles, irrespective of the type of pavement. Riding on concrete pavements increased the frequency of vibration events several times more than riding on asphalt pavements of equal distance [31]. Such safety concerns of e-scooter riders were investigated in 2019 through interviews conducted with 125 individuals [32]. Out of the riders interviewed, half expressed the belief that injuries were caused by surface conditions such as potholes or cracks on the pavement. Likewise, according to other studies, uneven pavement was cited by 10% of injured e-scooter riders as the cause of their accidents [33]. Regarding road type, a study in 2019 found that the majority of injuries (44%) occurred on sidewalks, and most victims had minor injuries [34]. We believe it is important for the government to make clear decisions on the designated areas for e-scooters to operate because the study highlights that sidewalks, often used by pedestrians, are the most common location for injuries. By defining specific zones for e-scooter use, the government can mitigate the risk of accidents and promote the safety of both e-scooter riders and pedestrians. In addition to these findings, it is important to consider the specific situation in Vilnius, Lithuania. The city’s old town is renowned for its charming pebble roads, which adds to its unique character but also presents a challenge for e-scooter riders. These old pebble roads can be uneven and prone to developing potholes over time, posing a significant risk to e-scooter riders. Furthermore, the city’s sidewalks, while essential for providing a safe pathway for pedestrians, can also be problematic for e-scooter riders due to cracks in the tiles. These cracks can cause instability and potentially lead to accidents and injuries to both riders and non-riders. This might be linked to one of the most significant findings of our study: the prevalence of injuries caused by tripping over uneven road surfaces, which accounted for a staggering 51.25% of all reported cases. Given the prevalence of these road conditions in Vilnius, city officials and urban planners must prioritize the maintenance and improvement of these surfaces to ensure the safety of all road users, including e-scooter riders.

Numerous governments, municipalities, institutions, and public entities have conducted comprehensive risk assessments associated with e-scooter operation and rental, leading to the implementation of preventive measures, including the outright prohibition of e-scooter sharing services or e-scooters in certain regions. The Netherlands exemplifies stringent regulations, where individuals operating e-scooters risk substantial fines [35]. Similarly, in 2018, Barcelona emerged as one of the pioneering cities to ban rental scooters from public roads [36]. The latest information indicates that Paris, setting a precedent in Europe, has completely banned e-scooter rental programs, while starting from 2023, e-scooters have been prohibited on all public platforms in neighboring Great Britain [37]. Reuters reports that Malta will enforce a comprehensive ban on all rental e-scooters effective March 1, 2024. On a global scale, several states in the United States have adopted comparable measures. San Francisco temporarily suspended e-scooters in June 2018, while West Hollywood and Beverly Hills instituted strict bans on e-scooters in 2018, prohibiting both scooter rentals and their operation within city limits. A similar scenario unfolded in Toronto, Canada. Across Asia, Singapore has prohibited e-scooter riding on sidewalks, and in South Korea, e-scooter riders must possess a relevant driver’s license, wear helmets, and are banned from riding on sidewalks. Notably, many institutions, such as university campuses in the United States and Great Britain, have also imposed bans on scooters. An incident on November 17, 2022, in Barcelona, involving an e-scooter explosion and subsequent fire on board a train, prompted the ban of e-scooters from public transport in both Barcelona and later in Madrid [38]. Furthermore, as of August 24, 2023, e-scooters are no longer permitted on the Hamburg subway.

The Lithuanian government, recognizing the escalating concerns, has implemented various restrictions on e-scooter operation and sharing in recent years. Notably, one significant change involved delineating the operational areas for shared e-scooter services. Drawing insights from global examples and leveraging our substantial dataset, we advocate for the consideration of additional restrictions and bans in Lithuania. Specific recommendations we endorse encompass mandatory helmet usage, potential bans or limitations on the existing large-scale e-scooter-sharing system, and the implementation of even more stringent speed limits. These measures are crucial for fostering a safer environment for both e-scooter riders and the general public, aligning with the evolving landscape of global regulations and prioritizing public safety.

We posit that the four-year longitudinal approach and the extensive dataset, offering valuable insights into the repercussions of prevalent e-scooter platforms and regulatory adjustments on injury trends, hold significance not only for Lithuania and the Baltic States but also for global considerations.

Study limitations

While our study provides valuable insights into e-scooter-related injuries in Vilnius, the generalizability of our conclusions to other urban environments may be limited. Future multicenter studies could provide a broader perspective. The documentation of alcohol use was often lacking in the majority of patients, making it challenging to draw definitive conclusions regarding actual alcohol involvement from our dataset. The retrospective design of the study prevented the establishment of causal relationships and limited the collection of data on non-patient-related characteristics, such as the time of day, day of the week, air temperature, wind speed, rain, visibility, and driving distance. Additionally, only a limited amount of data was available, encompassing aspects such as intoxication, helmet usage, and other protective measures (e.g., safety pads). Notably, no data on e-scooter speed during injuries was collected from 2018 to 2020. Furthermore, we were not granted permission to collect information on the financial burden resulting from trauma or the overall duration of hospitalization. The trauma numbers were influenced by regulations imposed during the coronavirus pandemic. Due to restrictions on work, travel, and social activities, there was a notable decrease in e-scooter usage, particularly in 2020. Patients with isolated face trauma, including fractures, were typically directed to another center, specifically the Vilnius University Hospital Žalgiris Clinic, which houses the Centre of Oral and Maxillo-Facial Surgery.

## Conclusions

The introduction of shared e-scooter services has precipitated a notable surge in e-scooter-related injuries, attributable to inadequate driver education, a lack of enforcement mechanisms, and uneven urban road conditions. Although a majority of e-scooter injuries manifest as mild, a substantial proportion assumes a moderate to severe nature, with certain cases culminating in fatality. Conducting further research in other Lithuanian regions would be beneficial to augment the existing study. To abate the incidence of e-scooter injuries reminiscent of those documented in other countries, a comprehensive array of preventive measures, encompassing rigorous road maintenance, speed reduction, mandatory helmet use, and utilization of other protective body gear, must be implemented. The government must delineate clear directives regarding the specified zones for e-scooter operations. Future studies should explore the impact of factors such as urban infrastructure changes, public awareness campaigns, and shifts in overall urban mobility habits to provide a more comprehensive understanding of the causes behind injury trends. We encourage replication of this research in varied settings to validate our findings and recommendations. Such studies can help tailor preventive measures to the specific needs and conditions of different cities.
